# Factors Influencing the Attendance of Preterm Infants to Neonatal Follow up And Early Intervention Services Following Discharge from Neonatal Intensive Care Unit during First Year of Life in Iran.

**Published:** 2018

**Authors:** Aida RAVARIAN, Roshanak VAMEGHI, Mohammad HEIDARZADEH, Shahin NARIMAN, Setareh SAGHEB, Fariba NORI, Farhoud SAEEDERSHADI, Mehdi NOROZI

**Affiliations:** 1 Arash Women ‘s Hospital, Tehran University of Medical Science, Tehran, Iran.; 2 2.Pediatric Neuro-rehabilitation Research Center, University of Social Welfare and Rehabilitation Sciences, Tehran, Iran.; 3 3.Pediatric Health Research Center, Tabriz University of Medical Sciences, Tabriz, Iran.; 4 4.Shariati Hospital, Tehran University of Medical Sciences, Tehran, Iran.; 5 5.Department of Orthotics and Prosthetics, University of Social Welfare and Rehabilitation Sciences, Tehran, Iran.; 6 Social determinants of Health Research Center, University of Social Welfare and Rehabilitation Sciences, Tehran, Iran.

**Keywords:** Preterm infant, Early intervention, Length of stay, Education, Awareness

## Abstract

**Objective:**

The aim of this study was to determine factors influencing the number of times neonatal intensive care unit admitted preterm infants attend Neonatal Follow up and Early Intervention services (NFEI) during first year of life.

**Materials &Methods:**

A parent-report questionnaire was administered via phone after the first birthday of preterm infants admitted to the NICU at Arash Hospital, Tehran, for at least 24 h, and who received standard NICU-based therapeutic services, from Apr 2014 to Feb 2015. Data included mother’s age, education, type of pregnancy, history of abortion or premature birth, self-reported post-partum depression, number of children, infant’s gender, birth weight, gestational age, length of stay in the NICU, living area, twin or triplet birth, number of siblings, and the child rank. Number of attending times to services was recorded. Another question addressed the causes of not attending the NFEI services.

**Results:**

Ultimately, 119 eligible children participated, 51% were girls and whose mean birth weight was 1908±626.7 gr, and average length of NICU stay was 20.1±16.9 d. After multivariate analysis, shorter length of stay in the NICU, lower maternal education, number of children, self-declared lack of awareness about early intervention services, and self-reported lack of referral by a physician were the only factors that continued to be significantly correlated, and in fact, the truly influential ones associated with number of attending times.

**Conclusion:**

This study has defined some predictors of poor follow up and early intervention service utilization in a high-risk group of infants suggested be addressing and tackling by policymakers.

## Introduction

Worldwide, more than 15 million babies are born prematurely every year and the rate of premature birth has risen in some countries (1). Premature infants are at risk of neurologic and developmental problems (2). Prematurity influences the child and the family and enforces large costs on healthcare systems. It can lead to about twice the cost of a term birth in the first 2 yr of life (3). Infants born preterm demonstrate developmental delay and behavioral problems in early childhood (4). Early developmental interventions are because plasticity in the developing brain is highest and thus developmental improvement in response to interventions is greatest early in life (5). If intervention begins earlier, starting therapy before 9 months of age will result in greater developmental progress (6).

Delay in accessing early intervention services can cause more severe developmental delays and higher rates of disabilities that will subsequently lead to more service demands and finally to higher costs, both for the families and the health care systems (7).

Early intervention after discharge from the neonatal intensive care unit (NICU) mainly concentrates on the infant’s development and parent-infant relationship (8). Early intervention in children born premature, leads to improved developmental and motor status (9). In many developed countries, premature infants are being routinely referred to receive early intervention services when discharged from the neonatal intensive care unit, since the 1980’s (10). However, in many other parts of the world in spite of the known advantages of early intervention services, unfortunately, many premature infants are still harmed due to delay in accessing early intervention (11). This is also true in Iran (12). 

There are various reasons for this delay in different societies such as low maternal education (13) and low professional knowledge about early intervention (14). Other factors reported to lead to low rates of registration for early intervention services are low referral rates from physicians and other professionals, parental shame (15), as well as, maternal depression (16), and low income (17). 

There is not enough data on the early intervention for developmental delays in Iran but many academic disciplines and policymakers have recently recognized and considered it. Since 4 yr ago, Tehran University of Medical Sciences, Tehran, Iran has established a neonatal follow-up and early intervention service (NFEI) for children <2 yr old with a history of prematurity, in Arash Hospital. This hospital is a women’s hospital located in the east of Tehran City, which also has a well-equipped neonatal ward and a specialized NICU. During the years that this hospital is providing early intervention services for premature infants, not all infants enrolled in this very important and needed program. 

The aim of this study was to define the possible existing barriers not well understood in Iran, which prohibit or hinder the utilization and taking advantage of this service by Iranian families. This will help relevant experts and policymakers to overcome possible barriers in order to maximize service utilization that will ultimately lead to higher developmental status in high-risk infants.

## Materials & Methods

Informed consent was obtained from the parents of all preterm infants participating in the study. The study was approved by Ethics Committee of the university.

This cross-sectional descriptive-analytical study used data from a cohort of preterm infants born in Arash Hospital, Tehran, Iran and admitted to the NICU at the same hospital from Apr 2014 to Feb 2015.

Data were gathered by completing a parent-report questionnaire administered via phone after the child’s first birthday. These included maternal characteristics such as age, education, type of pregnancy (natural or assistive), history of abortion, history of another premature child, history of self-reported post-partum depression and the number of children, as well as infant characteristics such as gender, birth weight, gestational age, length of stay in NICU, living area (Tehran city or its suburbs), twin or triplet birth, number of siblings, and the child rank. The parent-report questionnaire also included a close-ended question regarding the causes of attending or not attending the NFEI services, that Arash Hospital had provided for them. 

Utilization status of NFEI services was retrospectively tracked across the first year of the child’s life through medical records. 

Inclusion criteria included infants born ≤37 wk gestational age born in Arash Hospital and admitted to the same hospital’s NICU for at least 24 hours. Infants of a known congenital anomaly or suffered from severe sepsis or respiratory failure were excluded, because such infants were normally referred to other specialty hospitals immediately after diagnosis, so that they did not experience the standard NICU-based therapeutic services in this hospital, and were no longer easily accessible for following-up intervention services. 

All Infants who participated in our study had received standard care and NICU-based therapeutic services by a neonatal occupational therapist during their hospitalization period in the NICU. The therapist had also conducted parent education, had discussed the infant’s status with the parents and had recommended follow-up of therapy at discharge. In addition, the discharge process from the NICU had included providing all parents with an appointment time for receiving NFEI services.

**Statistical Analysis**

Through bivariate analysis using chi-square, we first investigated the association between infant and maternal factors and the number of times attending early intervention services. After checking for collinearity with a correlation matrix, variables that were marginally significant with a *P*-value<0.2 were included in the multiple linear regression models. Variables were eliminated from the multivariate models using stepwise selection. The final model included only variables with a *P*<0.05. All statistical analyses were conducted using SPSS-16 (Chicago, IL, USA)

## Results

**Fig 1:**
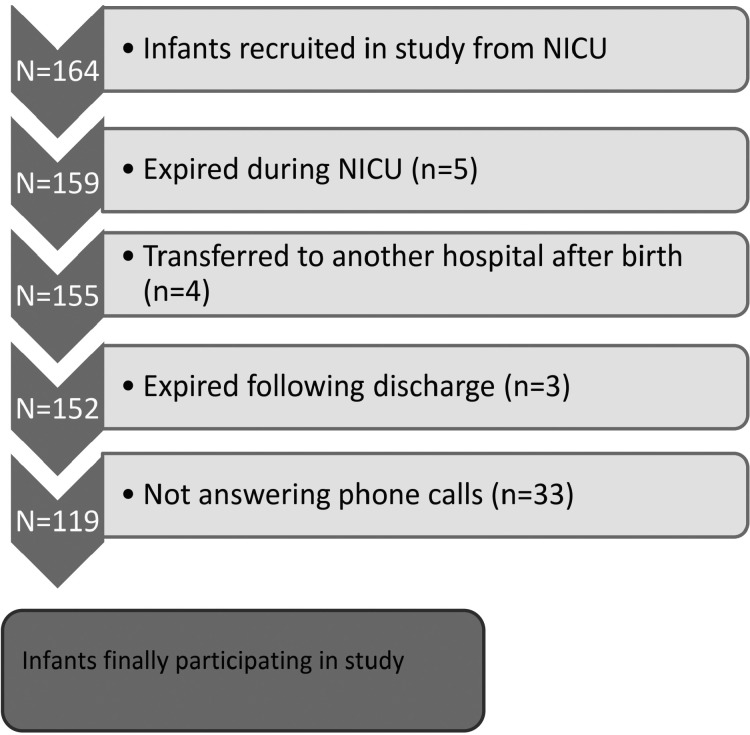
Flow diagram of infants excluded and finally included in study

Overall, 164 infants were enrolled. Five infants had expired during NICU hospitalization, four had been transferred to another hospital, three had expired following discharge, and 33 were later excluded due to not answering phone calls, leaving 119 eligible participants (Figure 1). There were no differences in key perinatal factors among those included and excluded.

Fifty-one percent of the babies were girls, 29% were born as twins and 46% were the first and only child. The mean birth weight of the infants was 1908 ± 626.7 gr. The average length of stay in the NICU was 20.1 d ± 16.9 SD. Overall, 65% of all participating premature children had returned and attended early intervention services before first birthday. Among children who ever attended, 20% attended the intervention sessions only one time, 10% attended two and 30% three times or more.

Mother’s education and number of children were the only maternal factors significantly associated with number of times attending (Table 2). In terms of infant factors, the baby’s weight and gestational age and the number of days hospitalized in the NICU, and in terms of self-reported causes of non-attendance, not being aware and not being referred by a physician, were the significantly correlated factors with number of times attending to follow-up early intervention services.

Variables that reached significance in bivariate linear regressions were then further evaluated in a multivariate regression model using a *P*≤0.05, in order to exclude confounding variables and to determine which variables were actually the true influential ones on number of attending times attendance to early intervention services. As demonstrated in Table 3, after multivariate analysis, shorter length of stay in the NICU (B: 0.01,*P*:0.04) lower maternal education (B: 0.26,*P*: 0.03), more number of children (B: -0.26, *P*:0.03), self-declared lack of awareness about NFEI services (B: -2.10,*P*: 0.00), and self-reported lack of referral by a physician (B: -0.57, *P*:0.02) were the only factors that were significantly correlated. The truly influential cases associated with number of tomes attending NFEI services.

## Discussion

The main factors associated with higher attendance to NFEI services after discharge from NICU were higher maternal education, longer length of stay in the NICU, less number of siblings, awareness about NFEI, and being referred by a physician. In the present study, more than one-third of the sample never returned to receive early intervention in the first year of their lives and among those who did return, only 35% attended three or more early intervention sessions. 

This rate of attendance is similar to that reported in several other studies conducted in Canada (18), Europe (19) and Australia (20) in which it was reported 10%-30%, and in the United States was 20% to 50% (21).

The present study showed that infants whose mothers had higher levels of education had a higher tendency to attend more NFEI sessions. The importance of women’s education on the health of their family and society is reported (22). Our results support the finding that women’s education and their knowledge have an important influence on higher rates of attendance of premature infants to FEI services and possibly better developmental outcomes for the child. One of the causes of correlation between college education and higher concern about the development of one’s child and higher intent to access intervention services is a higher rate of reading books about pregnancy and infants’ health by college-educated mothers (23). This may also be true in our study, but we did not investigate this issue.

In accordance with a study conducted in St Louise, Missouri (23), the mother‘s lack of awareness about early intervention was a significant variable for not attending follow-up services. Our findings showed that lack of awareness was a significantly correlated factor with number of times attendance to NFEI services took place. In our case, the discharge nurse had routinely appointed the next visit time for follow-up interventions and had explained the reason for this visit, to the mother. Therefore, this amount of explanation was not enough for raising the family’s awareness and intention to attend.

**Table 1:** Demographic and personal characteristics of participants

**Quantitative Variables(n=119)**	mean± SD	**Qualitative variables(n=119)**	%
**Infant factors** Gestational age (weeks) Birth weight (grams) Days in NCU	32.8 ± 2.5 1908 ± 626.7 20.1 ± 16.	**Infant factors** Twin or triplet birth(≥2) Singleton(having no siblings) female	32 46.2 51.03
**Maternal factors** Maternal age (years)	30.74±6.30	**Maternal factors** History of premature birth History of abortion High-school diploma and higher Assisted pregnancy Self-reported Depression	11.7 27.7 68 42 31.1
		**Home area** Tehran City Suburbs of Tehran City	71.4 28.6
		**Self-reported causes of not attending** Not being aware Not being referred by a physician Economic problems Felt not necessary Prohibition by members of the family No accessibility	31.9 27.7 18.5 18.5 17.6 21

**Table 2:** Correlation of all infant and maternal factors with number of times attending NFEI services by bivariate linear regression analysis

	**Variables**	**B**	**Std.Er**	***P*** **-value**
***Infant’s factors***	Sex *	-0.51	0.30	0.09*
	Gestational age*	-0.14	0.05	0.01*
	Weight *	-0.00	0.00	0.00*
	Days in NICU*	0.02	0.00	0.00*
	Born as twins	0.56	0.30	0.06
***Mother’s factors***	Mother’s age	0.03	0.02	0.17*
	Mother’s education*	0.39	0.16	0.02*
	Number of children*	-0.35	0.17	0.04*
	Pregnancy type	0.11	0.36	0.76
	Home area	-0.10	0.13	0.42
	History of premature baby	-0.34	0.33	0.30
	History of abortion	-0.16	0.13	0.23
	Self-reported depression	0.11	0.14	0.43
	Not being aware*	-2.51	0.23	0.00*
	Not being referred by a physician*	-1.33	0.30	0.00*
	Felt not necessary	-0.54	0.39	0.16*
	Family prohibition	0.17	0.40	0.67
	No accessibility	0.34	0.37	0.35
	Economic problems	0.28	0.39	0.46

* Remained significant after bivariate analysis (*P*≤ 0.2).

**Table 3:** Correlation of all infant and maternal factors with number of times attending follow-up and early intervention services by multivariate linear regression analysis

	Variable	B	Std. Err	*P*-value	95.0% Confidence Interval	
					Lower Limit	Upper Limit
Infant’s factors	Sex of baby	-0.14	0.20	0.47	-0.56	0.26
	Gestational age	0.08	0.07	0.23	-0.05	0.22
	Days in NICU*	0.01	0.00	0.04*	0.00	0.03
	Weight of baby	0.00	0.000	0.21	-0.00	0.00
	Twin	0.21	0.221	0.32	-0.21	0.65
**Mother’s factors**	Mother's age	0.00	0.01	0.8	-0.02	0.03
	Mother’s education*	0.26	0.12	0.03*	0.02	0.5
	Numbers of children*	-0.26	0.12	0.03*	-0.52	-0.02
	Not being aware*	-2.10	0.23	0.00*	-2.57	-1.61
	Not being referred by a physician*	-0.57	0.24	0.02*	-1.05	-0.09
	Felt not necessary	-0.27	0.26	0.29	-0.80	0.24

* Remained significant after multivariate analysis (*P* ≤ 0.05).

In this study, we did not find any significant correlation between maternal age and number of attending times. This finding is in accordance with another study (23), but in contrast to that, it was showed older mothers were more likely to submit to follow-up sessions (24). Our finding may be because the mothers participating in our study were not of a very wide age range and were all young.

Unlike what we had anticipated, history of abortion, and history of previously experiencing premature birth did not significantly affect number of attendance times. History of abortion or premature birth could act as a sensitizing and agent for the mother, to stimulate higher rates of care for the baby’s health and thus higher rates of attendance. The reason this turned out differently in our study is not clear. However, the results may have been different with a larger sample size.

In our sample, 42% of mothers reported some degree of feeling depressed after delivery. About 30%-40% of mothers of preterm infants experience post-partum depression. This is a rather high figure compared with the prevalence of depression in mothers of healthy term infants, which accounts for 6%-12% in some studies (25). However, we did not find significant difference in number of attending times to NFEI services between mothers who self-reported depression and anxiety after delivery and those who did not. Of course, we did not measure maternal depression by any valid tools and only relied on the mothers’ self-report. There are controversial findings on this matter. Maternal depression was associated with lower attendance in early intervention programs (16). However, children of mothers with depressive symptoms were 3 times more likely to participate in early intervention programs than others were (23, 26).

The finding that lower rates of attendance to early intervention services is related to having more children, remained as one of the most dominantly significant factors on multivariate analysis. Having more children reduces the time, power and concern for development that parents can provide for each child (27). Having more children produces a pseudo feeling of high-experience and confidence for the parents and the feeling of lack of necessity for any other interventions, even for the premature baby.

When analyzed with the bivariate regression model, the baby’s gestational age and birth weight, as well as the number of days hospitalized in the NICU were all significantly correlated with the number of times attendance had occurred. However, multivariate regression between these three variables showed that in contrast to some other studies reported significance of correlation between gestational age or birth weight (28) and attendance for early intervention services. The main factor affecting number of attendance times of infants to early intervention sessions in our study was actually the length of stay in the NICU, a variable which many other studies seem to have overlooked and had not considered in their research. The longer days the infant is hospitalized in the NICU, the higher the focus and concern of parents for their child will be. Moreover, spending more time in the NICU and directly observing the services that experienced personnel and specialists provide for the vulnerable premature infants may also have an impact on the parents’ attitude towards the importance and necessity of early intervention services administered by specialized personnel and experts for the better prognosis and future of their premature babies, which in turn may lead to more seriously attending FEI services. Yet another reason may be that parents of more severely ill infants who thus need longer NICU care feel stronger about the necessity of attending follow-up interventions. 

When self-reported economic problems were analyzed with multivariate regression in the present study, no associations were detected with number of attending times. This finding is analogous to that report associations between parents’ economic problems and their concern for the development of their child or for their intent to access therapy and. In contrast, financial hardship is a key barrier for accessing to clinical centers (24, 28, 29). In the participants of our study, who were mostly of an average or lower than average socio-economic class, financial shortcomings that most probably have existed for the parents, did not serve as a significant barrier for pursuing proper care and therapy for their infants, if necessary. 

About 27% of our participants mentioned that not being referred for following up early intervention services by a physician was the cause of not attending or their lower rates of attendance; this factor turned out to be a significantly correlated factor with number of attending times. This rather high figure of non-referrals by a physician may be due to different causes such as lack or insufficiency of professional knowledge or attitude regarding this issue and about its importance in many Iranian general physicians and pediatricians (12). Unlike many developed countries in which nearly most NICUs have follow-up clinics to which discharged infants are routinely referred (30), follow-up and early intervention for premature or other at-risk infants after discharge from the NICU are not practiced routinely in Iran and no protocols exist for practicing it. Thus many NICUs may not be providing such services and many physicians may not be aware of the existence of such services and its benefits to the child.

Some limitations of this study include it being an observational study with a rather small sample size for the number of variables included. This was inevitable because the sample was recruited from a census of all children who had the inclusion criteria. In addition, the Arash Woman’ Hospital cohort included a large number of infants of low socioeconomic status. Thus, our findings may not generalize to all preterm children aged zero to one year, across the country. 

**In conclusion, **we recommend that practitioners and policymakers consider factors as important and utilize this knowledge in detection of families and infants that may need more support than usual, as well as some different kinds of interventions for improving their timely and consistent utilization of early intervention services following discharge. We are hopeful that the results of this study have defined some predictors of poor follow-up and poor attendance to early intervention service utilization in a high-risk group of infants following NICU discharge. The potential causes identified in this study be addressed by practitioners and policymakers to enable better utilization and continuity of services that are aimed at optimizing attending to early intervention service after discharge from NICU for this vulnerable population of children. 
